# Causal relationship between genetic-predicted uric acid and cervical cancer risk: evidence for nutritional intervention on cervical cancer prevention

**DOI:** 10.3389/fnut.2024.1464046

**Published:** 2024-09-20

**Authors:** Chunge Cao, Dajun Cai, Hao Liu, Xia Zhang, Lina Cai, Caiping Sun, Huifang Wang, Hu Zhao, Chaoyan Yue

**Affiliations:** ^1^Department of Obstetrics and Gynecology, Second Affiliated Hospital of Zhengzhou University, Zhengzhou, China; ^2^Center for Reproductive Genetics and Prenatal Diagnosis, Hebi City People’s Hospital, Hebi, China; ^3^Department of Laboratory Medicine, Second Affiliated Hospital of Zhengzhou University, Zhengzhou, China; ^4^Department of Laboratory Medicine, Obstetrics and Gynecology Hospital of Fudan University, Shanghai, China

**Keywords:** uric acid, cervical cancer, Mendelian randomization, nutritional intervention, dietary management, cancer prevention

## Abstract

**Introduction:**

The relationship between serum uric acid (SUA) and cervical cancer is inconclusive. This study aims to investigate the causal relationship between SUA levels and cervical cancer incidence, and to evaluate the potential role of nutritional interventions in cervical cancer prevention.

**Methods:**

We conducted a two-sample bidirectional Mendelian randomization (MR) analysis using genetic instruments from publicly available genome-wide association studies (GWASs) of individuals of predominantly European ancestry. Methods such as inversevariance weighted, weighted-median, weighted model, and MR-Egger were applied. Sensitivity tests, including leave-one-out, MR-PRESSO, and Cochran’s Q test, assessed heterogeneity and pleiotropy.

**Results:**

Our findings revealed that a high SUA concentration significantly increased the risk of malignant cervical cancer: a 1 mg/mL increase in SUA was associated with a 71% higher risk (OR = 1.71, 95% CI = 1.10–2.67; *p* = 0.018). Stratification by histological type showed a significant causal effect on cervical adenocarcinoma risk (OR = 2.56, 95% CI = 1.14–5.73; *p* = 0.023). However, no clear evidence was found for a causal effect of cervical cancer on SUA levels.

**Conclusion:**

This study identified a causal relationship between elevated SUA levels and the risk of malignant cervical cancer, particularly cervical adenocarcinoma. These findings provide novel insights into the mechanisms of cervical carcinogenesis and suggest that managing SUA levels could be a potential strategy for cervical cancer prevention through dietary management.

## 1 Introduction

Cervical cancer is the fourth most common malignancy in terms of both incidence and mortality among females worldwide, causing more than half a million new cases and more than 300,000 deaths each year (1). The human papillomavirus (HPV) is essential but not sufficient for the transformation of cervical epithelial cells, indicating that there are other risk factors or protective factors involved (2). Therefore, exploring the pathogenesis and influencing factors of cervical cancer is highly important for its prevention and treatment.

Uric acid (UA) is the end product of human purine metabolism and is synthesized from hypoxanthine and xanthine by the action of the enzyme xanthine oxidoreductase. Approximately 98% of UA is ionized as urate and has a single negative charge at physiological pH (3). Nutritional choices that include a diet high in purines, such as red meat and seafood, along with other dietary factors that facilitate purine nucleotide degradation (e.g., alcohol and fructose consumption), can lead to elevated serum uric acid (SUA) concentrations (4). On the nutritional front, it has been observed that dietary intake of vitamin C is inversely associated with SUA levels, especially in men (5). At physiological concentrations, UA is a potent antioxidant that has anti-inflammatory and free radical scavenging effects (6, 7). However, at high concentrations, UA is a pro-oxidant molecule that contributes to free radical formation, resulting in oxidative cell damage, sterile inflammation, insulin resistance, and metabolic syndrome, which are all risk factors for cancer development (8). Some observational studies focused on the association between UA and cancer incidence risk have shown conflicting results, which indicates that the association May be sex-and site-specific (9–12). Moreover, the relationship between SUA levels and cervical cancer risk is still inconclusive (12, 13). However, these observational studies are at high risk of being confounded because factors related to UA levels, such as age, diet, diabetes status, kidney function and inflammation, May also be associated with cancer development (8). Moreover, an increase in UA levels May result from cancer-related cell renewal and apoptosis, indicating that reverse causality May also bias the relationship between UA and cancer risk (14).

Mendelian randomization (MR) is a novel epidemiological method in which genetic variants are used as instrumental variables to infer causal effects of exposures on outcomes (15). Genetic variants are independently isolated and randomly assigned during gamete formation and conception and are not affected by the onset or progression of the outcome (16). Therefore, MR is generally less prone to reverse causation or confounding and can strengthen causal inference compared with traditional observational methods (17). In this study, we used a two-sample bidirectional MR design to explore the potential associations of SUA with cervical cancer, assess the direction of association, and estimate its effect size, thereby offering scientific evidence for the role of nutritional intervention of uric acid concentrations in the prevention of cervical cancer.

## 2 Methods

### 2.1 Study design

This bidirectional two-sample MR study applied available datasets from large-scale genome-wide association (GWAS) studies to evaluate the causal effect of SUA levels on cervical cancer incidence as well as the causal effect of cervical cancer on SUA levels. Genetic variations [single-nucleotide polymorphisms (SNPs)] from the GWAS were used as instrumental variables for the exposure. The MR design is based on three important assumptions. First, genetic variation is strongly associated with exposure. Second, genetic variation is related only to outcome through the investigated exposure. Third, genetic variation is independent of any potential confounding factors (18). This study used publicly available summary-level GWAS data from published studies for which institutional review board approval was obtained. The data used in this study did not require additional ethical approval or informed consent, as they were public, anonymized, and de-identified.

### 2.2 Data sources

The summary-level data for UA were obtained from a genome-wide association study (GWAS ID: ieu-a-1055) conducted by the Global Urate Genetics Consortium (GUGC), available at https://gwas.mrcieu.ac.uk/datasets/ieu-a-1055/. Urate concentrations were measured in 110,347 individuals of European descent, encompassing 2,450,548 SNPs. The unit for urate concentration is mg/dl, primarily measured using the uricase method. Mean serum urate concentrations ranged from 3.9 to 6.1 mg/dL, with a median of 5.2 mg/dL. The prevalence of gout among these individuals ranged from 0.9 to 6.4%, with a median of 3.3%. Five datasets for cervical cancer were obtained from the FinnGen database:^1^ 1. Malignant neoplasm of uterus: cervix uteri (controls excluding all cancers, 167,558 European participants including 369 patients and 167,189 controls); 2. Squamous cell neoplasms and carcinoma of cervix (controls excluding all cancers, 167,353 European participants including 164 patients and 167,189 controls); 3. Adenocarcinomas of cervix (controls excluding all cancers, 167,301 European participants including 112 patients and 167,189 controls); 4. Other benign neoplasm of the uterus: cervix uteri (210,870 European participants including 146 patients and 210,724 controls); 5. Carcinoma *in situ* of cervix uteri (controls excluding all cancers, 167,637 European participants including 2,236 patients and 165,401 controls). The dataset of malignant neoplasm of uterus: cervix uteri included the following major histological types of cervical cancer: squamous carcinoma, adenocarcinoma, adenosquamous carcinoma, adenoid cystic carcinoma and undifferentiated carcinoma.

### 2.3 Instrumental variant selection

Genetic variants associated with urate levels were selected according to the threshold requirement of *p* < 5e-08 at the genome-wide significance level. To obtain enough SNPs that were associated with cervical cancer risk, a less stringent cut-off (*p* < 5e-05) was used. The parameters used to eliminate linkage disequilibrium among variables were clump distance >10,000 kb and r2 < 0.001, and the European ancestry data from the 1,000 Genomes Project (RRID: SCR_008801) were used as reference panel. To ensure the accuracy of the results, palindromic SNPs with intermediate allele frequencies were deleted (19). We searched the PhenoScanner V2 database^2^ to identify and remove any genetic variants associated with potential confounders of the outcomes. The F statistic was used to eliminate the bias caused by weak instrumental bias. The SNP-specific F-statistic was calculated by the square of the beta divided by the variance for the SNP-exposure association, and *F* < 10 was considered to indicate dubious bias (20).

### 2.4 Mendelian randomization analysis

We employed four types of MR analysis methods to estimate causal effects: the inverse variance-weighted (IVW) model, MR-Egger regression model, weighted-median estimator model, and weighted mode-based method. In the absence of directional pleiotropy, the IVW method can estimate causal effects more stably and accurately by combining the ratio estimates from each instrumental variant (21, 22). The MR-Egger regression method can test and adjust for directional pleiotropy and provide a consistent estimate of the causal effect that agrees with conventional MR methods; however, this method May be biased and have inflated Type 1 error rates in practice (23). The weighted-median estimator method is a robust method for causal inference that can handle up to 50% of invalid instrumental variables; this method yields lower Type 1 error rates and better adjustment for horizontal pleiotropy than does the inverse-variance weighted method and is complementary to the MR-Egger regression method (24). The weighted mode-based method is consistent when the largest number of similar individual-instrument causal effect estimates are from valid IVs, even if the majority of instruments are invalid, and when the instrumental variable assumptions are relaxed (25).

### 2.5 Sensitivity analysis

Cochran’s Q test was used to explore the heterogeneity between instrumental variables. A *p* value less than 0.05 indicated significant heterogeneity, and we applied a random effects model for the subsequent analyses. Otherwise, we used a fixed-effects model (26). Leave-one-out sensitivity analysis was applied to determine the reliability of the causal effect on a particular variant (27). The MR-Egger intercept test was used to detect and correct for bias due to directional pleiotropy, and an intercept term that differed from zero was used to indicate overall directional pleiotropy (28). The MR-PRESSO method was implemented to detect and correct for horizontal pleiotropy (29).

### 2.6 Statistical analysis

Analysis was performed using the two-sample MR method implemented in the package TwoSampleMR of R statistical software (v.4.3.1). Causal estimates are presented as odds ratios (ORs) and 95% confidence intervals. A two-tailed *p* < 0.05 was considered to indicate a significant difference.

## 3 Results

### 3.1 Genetically predicated effect of SUA levels on the risk of cervical cancer

**Figure 1:**
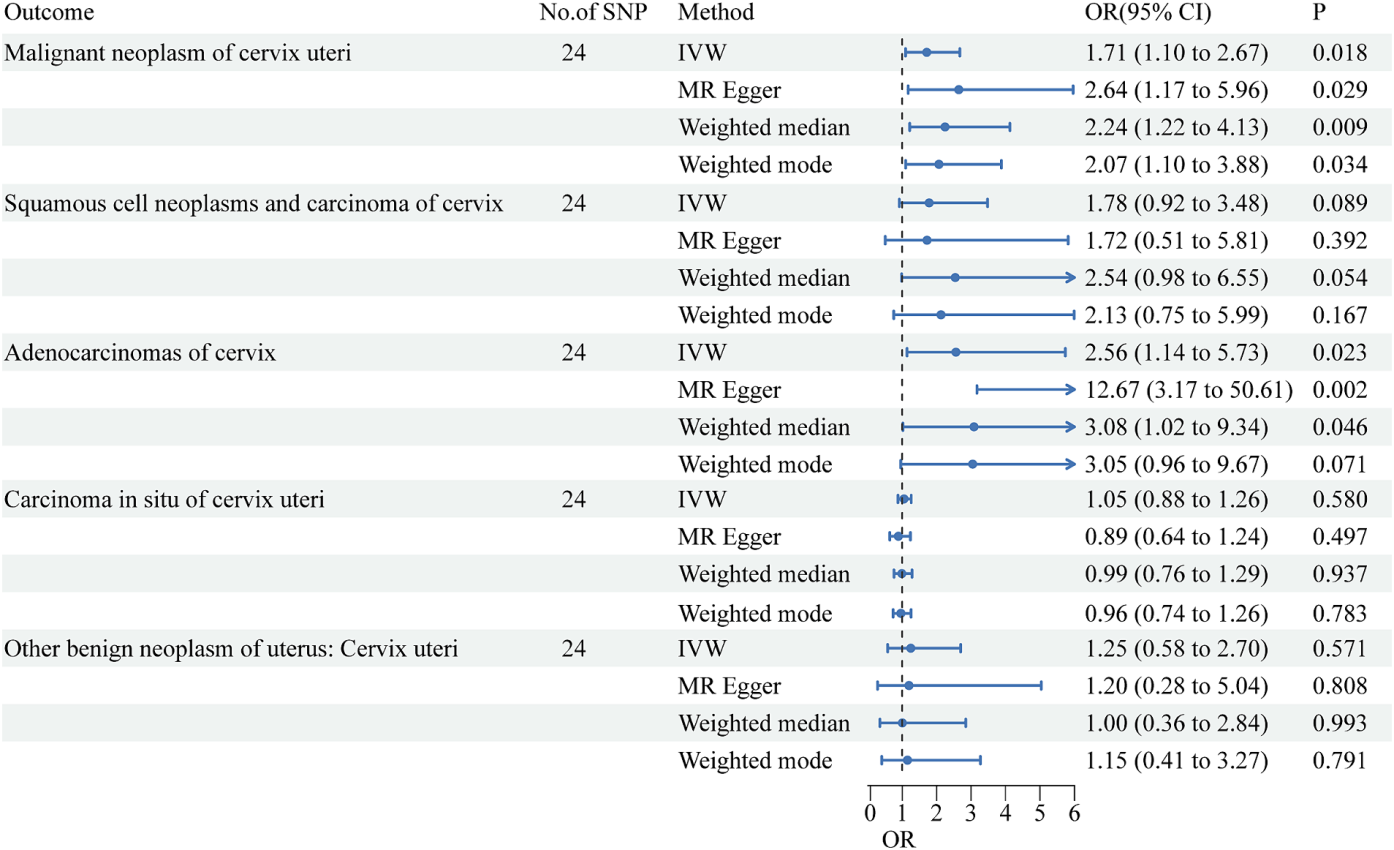
Forest plots show causal-effect estimates of per 1-mg/dl higher UA concentration on risks of cervical cancer subtypes. The results are shown for the different Mendelian randomization analysis methods used in this study. No., number; IVW, inverse variance weighted; OR, odds ratio; CI, confidence interval.

After screening for significance level and removing LD, we extracted 27 SNPs that were strongly associated with urate. We further found that 27 SNPs were shared by urate and the five cervical cancer subsets and that no SNPs were associated with cervical cancer or confounding factors. The SNPs rs1165151, rs17632159, and rs6830367 were removed because of the presence of a palindrome with a medium allele frequency. The remaining 24 SNPs were selected as instrumental variables in the analysis. The F-statistic ranged from 35.4 to 1406.3, indicating that weak instrument bias was not substantial. Details of the SNPs are presented in Supplementary Table S1. The detailed causal effect estimates of SUA levels on cervical cancer risk in this study are shown in Figure 1. The sensitivity analysis results are displayed in Table 1. The plots of the visualized data in this study, including the scatter plot, the forest plot, the leave-one-out analysis, and the funnel plot, are provided in Supplementary Figures S1–S5.

**Table 1:** Sensitivity analysis of the causal relationship between SUA levels and cervical cancer risk.

Outcome	Cochran’s *Q* test		MR-Egger test		MR-PRESSO test		
	*Q*	*p*-value	Intercept	*p*-value	RSSobs	*p*-value	Outliers
Malignant neoplasm of cervix uteri	13.893	0.930	−0.0393	0.224	15.2645	0.928	None
Squamous cell neoplasms and carcinoma of cervix	14.442	0.914	0.0033	0.945	16.2959	0.896	None
Adenocarcinomas of cervix	24.805	0.360	−0.1511	0.012	27.4863	0.334	None
Carcinoma *in situ* of cervix uteri	21.000	0.581	0.0152	0.247	22.6709	0.597	None
Other benign neoplasm of uterus: cervix uteri	27.293	0.244	0.0038	0.946	29.0336	0.285	None

A high SUA level had a significant causal relationship with malignant cervical neoplasms (IVW OR = 1.71, 95% CI = 1.10–2.67, *p* = 0.018). The results of MR-Egger (OR = 2.64, 95% CI = 1.17–5.96, *p* = 0.029), weighted median (OR = 2.24, 95% CI = 1.22–4.13, *p* = 0.009), and weighted mode (OR = 2.07, 95% CI = 1.10–3.88, *p* = 0.034) agreed with those of IVW. The MR-Egger intercept test indicated no occurrence of directional pleiotropy (intercept = −0.0393, *p* = 0.224). The MR-PRESSO test did not support any evidence of horizontal pleiotropy or outliers (global test *p* value = 0.928).

A high SUA level had a significant causal relationship with cervical adenocarcinoma (IVW OR = 2.56, 95% CI = 1.14–5.73; *p* = 0.023). The results were replicated via MR-Egger (OR = 12.67, 95% CI = 3.17–50.61, *p* = 0.002) and weighted median analysis (OR = 3.08, 95% CI = 1.02–9.34, *p* = 0.046). The associations were directionally similar according to the weighted mode method (OR = 3.05, 95% CI = 0.96–9.67; p = 0.023). The MR-Egger intercept test indicated that pleiotropic SNPs May be present (intercept = −0.1511, *p* = 0.012), but MR-PRESSO did not identify any outliers.

High SUA levels were positively associated with the risk of cervical squamous cell carcinoma, although the results were not statistically significant (IVW OR = 1.78, 95% CI = 0.92–3.48; *p* = 0.089). All other three MR methods yielded results consistent with those of the IVW method. The MR-Egger intercept test did not support any evidence for directional pleiotropy (intercept = 0.0033, *p* = 0.945). The MR-PRESSO test provided no evidence of horizontal pleiotropy or outliers (global test *p* value = 0.595).

The SUA concentration was not associated with the risk of cervical carcinoma *in situ* (IVW OR = 1.05, 95% CI = 0.88–1.26, *p* = 0.580) or with benign cervical neoplasm (OR = 1.25, 95% CI = 0.58–2.70, *p* = 0.571). Cochran’s *Q* test showed that there was no sign of heterogeneity across the individual effect estimates derived from every SNP in this MR study. The robustness of our results was confirmed by the leave-one-out sensitivity test.

Genetically predicated effect of cervical cancer on SUA levels.

**Figure 2:**
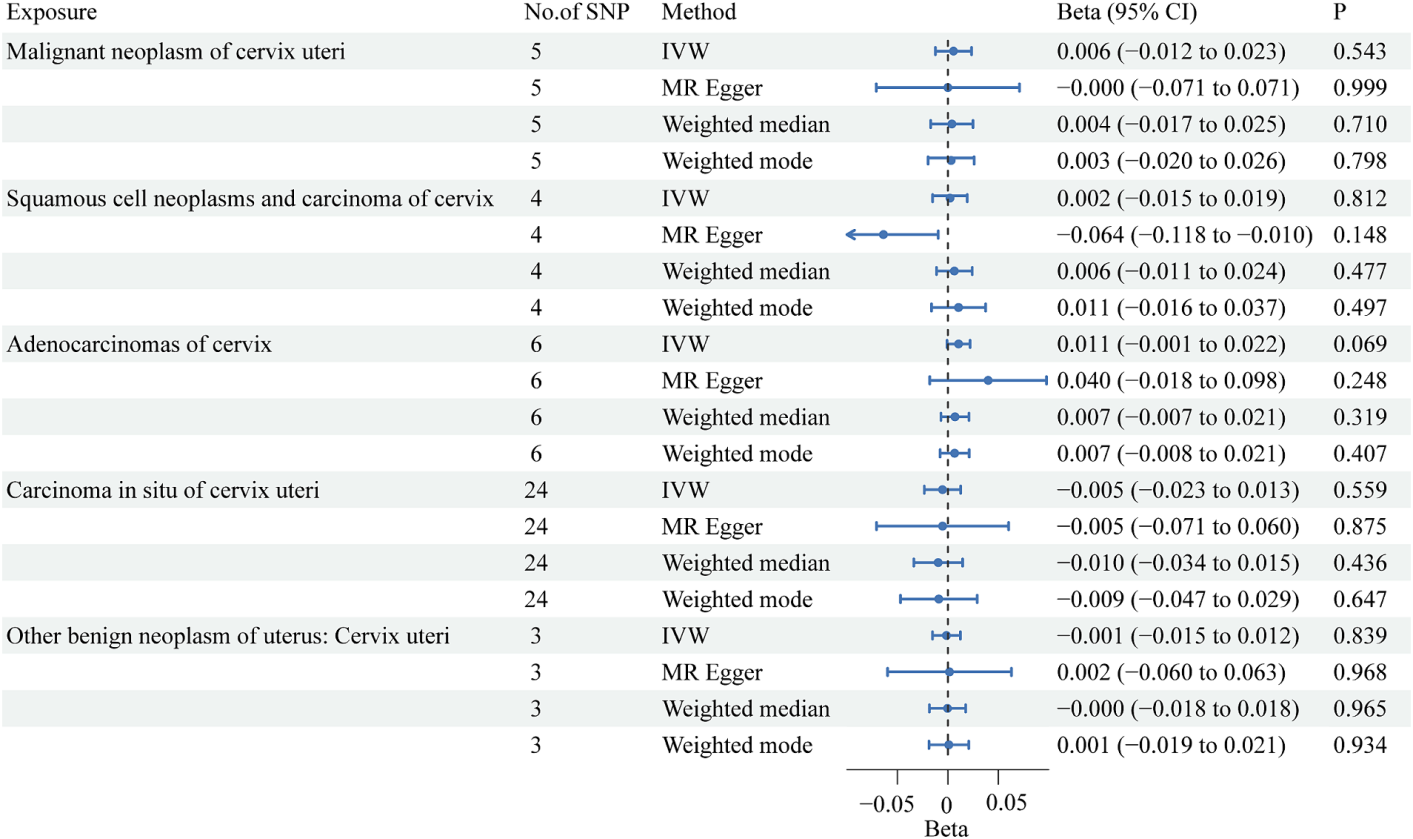
Forest plots showing causal effects of cervical cancer subtypes on SUA levels. The results are shown for the different Mendelian randomization analysis methods used in this study. No., number; IVW, inverse variance weighted; CI, confidence interval.

Among the five cervical cancer datasets, all had 3 or more independent genome-wide significant SNPs according to the less stringent cut-off (*p* < 5e-05). The F-statistic ranged from 16.5 to 26.6, indicating that weak instrument bias was not substantial. Details of the SNPs used in the analysis are presented in Additional file: Supplementary Tables S2–S6. The detailed causal effect estimates of cervical cancer incidence on SUA levels in this study are shown in Figure 2. The sensitivity analysis results are displayed in Table 2.

**Table 2:** Sensitivity analysis of the causal relationship between cervical cancer and SUA level.

Exposure	Cochran’s *Q* test		MR-Egger test		MR-PRESSO test		
	*Q*	*p*-value	Intercept	*p*-value	RSSobs	*p*-value	Outliers
Malignant neoplasm of cervix uteri	0.305	0.990	0.0020	0.884	0.4411	0.992	None
Squamous cell neoplasms and carcinoma of cervix	6.602	0.090	0.0346	0.135	14.0629	0.178	None
Adenocarcinomas of cervix	5.460	0.362	0.0184	0.367	6.6540	0.514	None
Carcinoma *in situ* of cervix uteri	23.147	0.452	0.0152	0.247	29.0336	0.285	None
Other benign neoplasm of uterus: cervix uteri	1.428	0.450	0.0000	0.998	NA	NA	NA

NA, not applicable.

The IVW results showed that none of the five histological subtypes of cervical cancer were causally related to the SUA level. The results of the other three MR methods were consistent with the IVW results. Both the MR-Egger intercept test and the MR-PRESSO test results showed no potential for pleiotropy. The MR-PRESSO test could not be applied for sensitivity analysis of the causal relationship between benign cervical neoplasms and SUA levels because there were no more than 3 SNPs. Cochran’s Q test indicated that there was no sign of heterogeneity in the SNPs. The robustness of our results was confirmed by the leave-one-out sensitivity test.

## 4 Discussion

This two-sample bidirectional MR study showed that high SUA levels had a significant causal effect on malignant cervical cancer risk. Further analysis of the histological subtypes indicated that high SUA levels were causally associated with an increased risk of cervical adenocarcinoma, and positively associated with the risk of cervical squamous cell carcinoma without statistical significance. The reverse MR analysis showed no evidence of a causal effect of cervical cancer on SUA levels. These findings provide a scientific basis for the role of nutritional intervention of uric acid concentration in the prevention of cervical cancer.

A large number of observational studies have demonstrated a positive correlation between SUA levels and cancer incidence, although the correlation seems to vary by sex and cancer site. SUA levels are positively associated with the incidence of kidney cancer, especially in female subjects (10). A positive correlation between high SUA and cancer incidence appears to be more significant in females with pancreatic cancer, while in male with gallbladder cancer (11). Gout was significantly associated with increased cancer incidence, especially for esophageal, stomach, colon, liver, pancreatic, lung, ovarian, renal, and bladder cancers (12). One study utilized six machine learning classifiers to analyze pretherapeutic serological parameters in early-stage cervical cancer patients, finding that blood markers, including uric acid and D-dimer, are associated with pathologic risk factors, suggesting a potential link between elevated uric acid levels and cervical cancer (30).There are several potential mechanisms that May explain the association between high UA levels and increased cancer risk. First, high levels of UA May induce inflammatory stress responses and stimulate various transcription factors that promote cell proliferation and migration, turning normal resting cells into highly aggressive cancer cells (14). Second, ROS are produced as by-products when xanthine oxidoreductase converts xanthine into UA (31). Therefore, high UA levels May disrupt the balance between ROS generation and elimination, which affects the normal redox state in normal cells and facilitates the initiation of tumorigenesis (32). Third, as “two-faced” molecules, ROS are also involved in triggering apoptosis. The ROS scavenging properties of UA May protect cancer cells from oxidative stress-induced apoptosis, thereby promoting their growth and survival (33).

Studies have reported that elevated SUA levels are associated with a decreased risk of cancer incidence and mortality, supporting the hypothesis that UA plays a protective role in cancer development due to its antioxidant property (9, 34). However, a systematic review and meta-analysis revealed no significant association between UA levels and cancer incidence (35). The conflicting results concerning the association between high UA levels and cancer risk derived from these epidemiological studies May be attributed to variations in study design, sample size, source of controls, cancer types, genetic background and other factors.

One MR study evaluated the relationship between SUA and cancer incidence and all-cause mortality and revealed that a genetically determined 50% higher SUA was associated with a 22% increased risk of cancer incidence (OR = 1.22, 95% CI = 1.02–1.47) and a 49% increased risk of all-cause mortality (OR = 1.49, 95% CI = 1.13–1.93). However, other MR studies did not find any significant causal effect of SUA levels on the risk of eight site-specific cancers, such as bladder, breast, colorectal, lung, prostate, renal cell, skin, and thyroid cancers (36, 37); or digestive system cancers, including esophageal, stomach, colon, pancreatic, and liver cancers (38). These MR studies confirmed that UA May have different effects on carcinogenesis depending on the sex, site or cell type of the cancer.

The cervix is composed of two types of epithelium: stratified squamous epithelium that covers the exocervix and mucus-secreting columnar epithelium that lines the endocervical canal. Tumors derived from the ectocervix are mostly squamous cell carcinomas, which account for approximately 75% of invasive cervical carcinoma cases. On the other hand, tumors arising from the endocervix are more likely to be adenocarcinomas. Other less common histological subtypes of cervical carcinoma include adenosquamous, small cell or neuroendocrine, serous papillary, and clear cell carcinomas (39). Our study was the first MR study to evaluate the causal association between SUA levels and the risk of cervical cancer according to histological subtype. We found that high UA levels had a causal effect on malignant cervical neoplasms. When we further examined the cervical cancer histological subtypes, we found that SUA levels had a causal relationship with cervical adenocarcinoma but were positively associated with the risk of cervical squamous cell carcinoma without statistical significance. We speculated that the different effects of SUA levels on cervical adenocarcinoma and squamous cell carcinoma might be due to the different distributions of UA in the extracellular environment of the exocervix and endocervical canal or variations in cellular responses to UA stress in stratified squamous epithelium and mucus-secreting columnar epithelium. However, the underlying mechanism of this phenomenon requires further investigation.

The MR-Egger, weighted-median, and weighted mode methods have less power to detect causal effects than does the IVW method, as evidenced by the wide confidence intervals (40), and they serve only as Supplementary materials in this study. When evaluating the causal effect of UA on cervical adenocarcinoma risk, the MR-Egger intercept test indicated the occurrence of directional pleiotropy, which could bias the causal estimate. However, the MR-Egger method gave a consistent causal estimate with the IVW method, although with a larger magnitude and wider confidence intervals. The ORs of MR-Egger, weighted-median, and weighted-mode were all greater than 1, indicating a positive association between high UA levels and cervical adenocarcinoma risk. Moreover, no heterogeneity, horizontal pleiotropy or outliers were detected, which supported the validity of the causal inference. Therefore, the results suggested a reliable causal relationship between high UA levels and cervical adenocarcinoma risk.

Eugenia pyriformis, a plant from the Myrtaceae family, possesses properties that can lower uric acid levels and inhibit the proliferation of HeLa cells, a type of cervical cancer cell (41). This suggests that reducing uric acid levels might help decrease the risk and progression of cervical cancer. A multifaceted nutritional strategies can contribute to lowering uric acid levels. Hydration plays a pivotal role, with sufficient water intake promoting uric acid excretion (4). Dietary modifications, specifically the reduction of purine-rich foods, directly correlate with decreased uric acid synthesis (4). The inclusion of vitamin C-rich fruits and vegetables not only enhances the diet’s antioxidant profile but also contributes to uric acid reduction (42). Moreover, the consistent consumption of dairy products appears to offer a protective effect due to specific milk proteins that aid in lowering uric acid concentrations (42). While the moderate intake of coffee provides beneficial antioxidants such as caffeine and chlorogenic acid, which are implicated in the reduction of uric acid levels, the consumption of alcohol and fructose should be curtailed, given their propensity to elevate uric acid (43). Lastly, an increased intake of alkaline foods and a decreased consumption of acidic foods May assist in maintaining the body’s acid–base homeostasis, further influencing uric acid levels (43). These nutritional interventions could serve as potential strategies for the prevention of cervical cancer. Moreover, a thorough investigation into additional nutritional factors that influence uric acid levels will provide more precise guidance for nutritional interventions aimed at preventing cervical cancer.

Our study has several important strengths. First, to the best of our knowledge, this study was the first MR analysis to investigate the causal relationship between SUA levels and cervical cancer histological subtypes, which significantly advanced the existing knowledge. Second, unlike previous epidemiological studies that examined only the association between UA and cancer risk, our MR design was less prone to confounding factors because we used multiple UA-associated SNPs from large-scale GWAS data as instrumental variables, which provided sufficient statistical power to infer causality. Third, we applied strict inclusion criteria for instrumental variables to satisfy the core assumptions of MR and avoid potential weak instrument bias, and we also used the MR-Egger and MR-PRESSO methods to detect and adjust for any deviations caused by horizontal or directional pleiotropy. In addition, we restricted the genetic background of the participants to mainly European ancestry to avoid potential confounding from a more heterogeneous population.

However, our study has several limitations. First, we focused on datasets with participants who were mainly of European descent, which might limit the generalizability of our results to other ethnic groups. Second, the publicly available GWAS data for UA did not report the specific features of UA, such as UA concentrations. Therefore, we could not further categorize UA or conduct a stratified MR analysis based on UA levels. Third, our MR analysis was based on publicly available summary-level data rather than individual data, which prevented us from testing the nonlinear causal relationship between UA levels and cervical cancer risk.

## 5 Conclusion

This bidirectional MR study demonstrated that elevated SUA levels significantly increase the risk of cervical adenocarcinoma. Additionally, a positive correlation with cervical squamous cell carcinoma was identified, although it did not achieve statistical significance. These findings highlight the critical need to monitor and manage elevated SUA levels as a component of cervical cancer prevention strategies, particularly in women at high risk. Additionally, our research supports incorporating SUA levels into cervical cancer risk prediction models, potentially improving public health guidelines and nutritional interventions. Future studies are warranted to elucidate the biological mechanisms underlying these correlations, providing a scientific foundation for tailored nutritional recommendations and public health initiatives.

## Data Availability

The original contributions presented in the study are included in the article/Supplementary material, further inquiries can be directed to the corresponding author/s.

## Footnotes

^1^ https://r9.finngen.fi/

^2^ http://www.phenoscanner.medschl.cam.ac.uk/

## References

[1] Bray F Ferlay J Soerjomataram I Siegel RL Torre LA Jemal A. Global cancer statistics 2018: GLOBOCAN estimates of incidence and mortality worldwide for 36 cancers in 185 countries. CA Cancer J Clin. (2018) 68:394–424. doi: 10.3322/caac.21492, PMID: 30207593

[2] Einstein MH Schiller JT Viscidi RP Strickler HD Coursaget P Tan T. Clinician’s guide to human papillomavirus immunology: knowns and unknowns. Lancet Infect Dis. (2009) 9:347–56. doi: 10.1016/S1473-3099(09)70108-2, PMID: 19467474

[3] Ruilope LM Juan GP. Hyperuricemia and renal function. Cardiovasc Risk Hypertens. (2001) 3:197–202. doi: 10.1007/s11906-001-0038-2 11353569

[4] Dalbeth N Gosling AL Gaffo A Abhishek A. Gout. Lancet. (2021) 397:1843–55. doi: 10.1016/S0140-6736(21)00569-9 33798500

[5] Yang Y Zheng S Feng Y. Associations between vitamin C intake and serum uric acid in US adults: Findings from National Health and nutrition examination survey 2011–2016. PLoS One. (2023) 18:e0287352. doi: 10.1371/journal.pone.0287352 37831704 PMC10575504

[6] Ames BN Cathcart R Schwiers E Hochstein P. Uric acid provides an antioxidant defense in humans against oxidant-and radical-caused aging and cancer: a hypothesis. Proc Natl Acad Sci U S A. (1981) 78:6858–62. doi: 10.1073/pnas.78.11.6858, PMID: 6947260 PMC349151

[7] Waring WS Convery A Mishra V Shenkin A Webb DJ Maxwell SRJ. Uric acid reduces exercise-induced oxidative stress in healthy adults. Clin Sci. (2003) 105:425–30. doi: 10.1042/CS20030149, PMID: 12801243

[8] Allegrini S Mercedes GG Pesi R Camici M Tozzi MG. The good, the bad and the new about uric acid in Cancer. Cancers (Basel). (2022) 14:4959. doi: 10.3390/cancers14194959 36230882 PMC9561999

[9] Taghizadeh N Vonk JM Boezen HM. Serum uric acid levels and cancer mortality risk among males in a large general population-based cohort study. Cancer Causes Control. (2014) 25:1075–80. doi: 10.1007/s10552-014-0408-0, PMID: 24906474 PMC4082647

[10] Dai XY He QS Jing Z Yuan JQ. Serum uric acid levels and risk of kidney cancer incidence and mortality: a prospective cohort study. Cancer Med. (2020) 9:5655–61. doi: 10.1002/cam4.3214, PMID: 32537937 PMC7402822

[11] Huang CF Huang JJ Mi NN Lin YY He QS Lu YW. Associations between serum uric acid and hepatobiliary-pancreatic cancer: a cohort study. World J Gastroenterol. (2020) 26:7061–75. doi: 10.3748/wjg.v26.i44.7061, PMID: 33311950 PMC7701939

[12] Oh YJ Lee YJ Lee E Park B Kwon J-W Heo J. Cancer risk in Korean patients with gout. Korean J Intern Med. (2022) 37:460–7. doi: 10.3904/kjim.2020.259, PMID: 32872748 PMC8925955

[13] Basu J Mikhail MS Ahn CW Furguiele J Ho GY Burk RD. Plasma uric acid levels in women with cervical intraepithelial neoplasia. Nutr Cancer. (2005) 51:25–31. doi: 10.1207/s15327914nc5101_4, PMID: 15749626

[14] Fini MA Elias A Johnson RJ Wright RM. Contribution of uric acid to cancer risk, recurrence, and mortality. Clin Transl Med. (2012) 1:16. doi: 10.1186/2001-1326-1-16 23369448 PMC3560981

[15] Bowden J Holmes MV. Meta-analysis and Mendelian randomization: a review. Res Sythesis Methods. (2019) 10:486–96. doi: 10.1002/jrsm.1346, PMID: 30861319 PMC6973275

[16] Smith GD Hemani G. Mendelian randomization: genetic anchors for causal inference in epidemiological studies. Hum Mol Genet. (2014) 23:R89–98. doi: 10.1093/hmg/ddu328, PMID: 25064373 PMC4170722

[17] Smith GD Ebrahim S. ‘Mendelian randomization’: can genetic epidemiology contribute to understanding environmental determinants of disease? Int J Epidemiol. (2003) 32:1–22. doi: 10.1093/ije/dyg070, PMID: 12689998

[18] Lawlor DA Harbord RM Sterne JAC Timpson N Smith GD. Mendelian randomization: using genes as instruments for making causal inferences in epidemiology. Stat Med. (2008) 27:1133–63. doi: 10.1002/sim.3034, PMID: 17886233

[19] Hartwig FP Davies NM Hemani G Smith GD. Two-sample Mendelian randomization: avoiding the downsides of a powerful, widely applicable but potentially fallible technique. Int J Epidemiol. (2016) 45:1717–26. doi: 10.1093/ije/dyx028, PMID: 28338968 PMC5722032

[20] Bowden J Fabiola Del Greco M Minelli C Smith GD Sheehan NA Thompson JR. Assessing the suitability of summary data for two-sample mendelian randomization analyses using MR-egger regression: the role of the I2 statistic. Int J Epidemiol. (2016) 45:1961–74. doi: 10.1093/ije/dyw220, PMID: 27616674 PMC5446088

[21] Burgess S Scott RA Timpson NJ Smith GD Thompson SG. Using published data in Mendelian randomization: a blueprint for efficient identification of causal risk factors. Eur J Epidemiol. (2015) 30:543–52. doi: 10.1007/s10654-015-0011-z, PMID: 25773750 PMC4516908

[22] Burgess S Dudbridge F Thompson SG. Combining information on multiple instrumental variables in Mendelian randomization: comparison of allele score and summarized data methods. Stat Med. (2016) 35:1880–906. doi: 10.1002/sim.6835 26661904 PMC4832315

[23] Burgess S Thompson SG. Interpreting findings from Mendelian randomization using the MR-egger method. Eur J Epidemiol. (2017) 32:377–89. doi: 10.1007/s10654-017-0255-x, PMID: 28527048 PMC5506233

[24] Bowden J Smith GD Haycock PC Burgess S. Consistent estimation in Mendelian randomization with some invalid instruments using a weighted median estimator. Genet Epidemiol. (2016) 40:304–14. doi: 10.1002/gepi.21965, PMID: 27061298 PMC4849733

[25] Hartwig FP Smith GD Bowden J. Robust inference in summary data Mendelian randomization via the zero modal pleiotropy assumption. Int J Epidemiol. (2017) 46:1985–98. doi: 10.1093/ije/dyx102, PMID: 29040600 PMC5837715

[26] Del GMF Minelli C Sheehan NA Thompson JR. Detecting pleiotropy in Mendelian randomisation studies with summary data and a continuous outcome. Stat Med. (2015) 34:2926–40. doi: 10.1002/sim.6522 25950993

[27] Hu B He X Li F Sun Y Sun J Feng L. Childhood obesity and hypertension in pregnancy: a two-sample Mendelian randomization analysis. J Hypertens. (2023) 41:1152–8. doi: 10.1097/HJH.0000000000003442, PMID: 37074353 PMC10241434

[28] Bowden J Smith GD Burgess S. Mendelian randomization with invalid instruments: effect estimation and bias detection through egger regression. Int J Epidemiol. (2015) 44:512–25. doi: 10.1093/ije/dyv080, PMID: 26050253 PMC4469799

[29] Verbanck M Chen CY Neale B Do R. Detection of widespread horizontal pleiotropy in causal relationships inferred from Mendelian randomization between complex traits and diseases. Nat Genet. (2018) 50:693–8. doi: 10.1038/s41588-018-0099-7, PMID: 29686387 PMC6083837

[30] Ou Z Mao W Tan L Yang Y Liu S Zhang Y. Prediction of postoperative pathologic risk factors in cervical Cancer patients treated with radical hysterectomy by machine learning. Curr Oncol. (2022) 29:9613–29. doi: 10.3390/curroncol29120755, PMID: 36547169 PMC9776916

[31] Battelli MG Polito L Bortolotti M Bolognesi A. Xanthine oxidoreductase in cancer: more than a differentiation marker. Cancer Med. (2016) 5:546–57. doi: 10.1002/cam4.601, PMID: 26687331 PMC4799950

[32] Tong LY Chuang CC Wu SY Zuo L. Reactive oxygen species in redox cancer therapy. Cancer Lett. (2015) 367:18–25. doi: 10.1016/j.canlet.2015.07.008 26187782

[33] Seifried HE Anderson DE Fisher EI Milner JA. A review of the interaction among dietary antioxidants and reactive oxygen species. J Nutr Biochem. (2007) 18:567–79. doi: 10.1016/j.jnutbio.2006.10.007 17360173

[34] Hsueh CY Shao MX Cao WJ Li SJ Zhou L. Pretreatment serum uric acid as an efficient predictor of prognosis in men with laryngeal squamous cell cancer: a retrospective cohort study. Oxidative Med Cell Longev. (2019) 2019:1–12. doi: 10.1155/2019/1821969 PMC6501142 31178950

[35] Dovell F Boffetta P. Serum uric acid and cancer mortality and incidence: a systematic review and meta-analysis. Eur J Cancer Prev. (2018) 27:399–405. doi: 10.1097/CEJ.0000000000000440, PMID: 29570104

[36] Horsfall LJ Hall IP Nazareth I. Serum urate and lung cancer: a cohort study and Mendelian randomization using UK biobank. Respir Res. (2021) 22:179. doi: 10.1186/s12931-021-01768-y, PMID: 34134711 PMC8210393

[37] Jiang MX Ren LJ Chen SZ Li GH. Serum uric acid levels and risk of eight site-specific cancers: a Mendelian randomization study. Front Genet. (2021) 12:608311. doi: 10.3389/fgene.2021.608311, PMID: 33767728 PMC7985250

[38] Zhang XN Zhao H Man JY Yin XL Zhang TC Yang XR. Investigating causal associations of diet-derived circulating antioxidants with the risk of digestive system cancers: a Mendelian randomization study. Nutrients. (2022) 14:3237. doi: 10.3390/nu14153237, PMID: 35956413 PMC9370260

[39] Jr WS Bacon MA Bajaj A Chuang LT Fisher BJ Harkenrider MM. Cervical cancer: a global health crisis. Cancer. (2017) 123:2404–12. doi: 10.1002/cncr.30667 28464289

[40] Slob EAW Burgess S. A comparison of robust Mendelian randomization methods using summary data. Genet Epidemiol. (2020) 44:313–29. doi: 10.1002/gepi.22295, PMID: 32249995 PMC7317850

[41] De Paula AT Toledo Martins Pereira M Sardou Charret T César Thurler Júnior J Freimann Wermelinger G Regina Baptista A. Evaluation of the Antiproliferative potential of Eugenia pyriformis leaves in cervical Cancer cells. Chem Biodivers. (2022) 19:e202200114. doi: 10.1002/cbdv.202200114 35798670

[42] Zhou M Huang X Li R Zhang Z Zhang L Gao X. Association of dietary patterns with blood uric acid concentration and hyperuricemia in northern Chinese adults. Nutr J. (2022) 21:42. doi: 10.1186/s12937-022-00789-7, PMID: 35739563 PMC9219223

[43] Mayo Clinic Staff. *Gout diet: What’s allowed, what’s not*. (2022). Available at: https://www.mayoclinic.org/healthy-lifestyle/nutrition-and-healthy-eating/in-depth/gout-diet/art-20048524.

