# Supplementary material for: Causal relationship between genetic-predicted uric acid and cervical cancer risk: evidence for nutritional intervention on cervical cancer prevention
Source: Front Nutr. 2024 Sep 20;11:1464046. doi: 10.3389/fnut.2024.1464046 (PMC11449874; doi:10.3389/fnut.2024.1464046)
Supplement: Supplementary file 1 [file Table_1.DOCX]

Supplementary materials

**Table S1.** List of SNPs used as instrumental variables for urate.

| SNP | Chr: Position | EA | OA | EAF | *F*-Statistic | Association with exposure | | | Association with outcome | | |
| --- | --- | --- | --- | --- | --- | --- | --- | --- | --- | --- | --- |
|  |  |  |  |  |  | Beta | SE | *P*-value | Beta | SE | *P*-value |
| rs11264341 | 1:155151493 | T | C | 0.412 | 66.188 | -0.048 | 0.006 | 1.04E-14 | -0.103 | 0.112 | 0.35 |
| rs1471633 | 1:145723739 | C | A | 0.493 | 127.606 | -0.061 | 0.005 | 1.40E-26 | -0.182 | 0.113 | 0.11 |
| rs1260326 | 2:27730940 | C | T | 0.410 | 196.000 | -0.077 | 0.006 | 1.31E-40 | 0.028 | 0.117 | 0.81 |
| rs17050272 | 2:121306440 | A | G | 0.450 | 36.791 | 0.037 | 0.006 | 9.36E-09 | -0.097 | 0.112 | 0.39 |
| rs2307394 | 2:148716428 | C | T | 0.325 | 37.704 | 0.035 | 0.006 | 7.26E-09 | -0.076 | 0.117 | 0.52 |
| rs6770152 | 3:53100214 | T | G | 0.414 | 73.469 | -0.048 | 0.006 | 2.66E-16 | -0.057 | 0.114 | 0.61 |
| rs11722228 | 4:9915741 | T | C | 0.346 | 1406.250 | 0.210 | 0.006 | 1.00E-200 | 0.212 | 0.119 | 0.07 |
| rs1825043 | 4:9573740 | A | G | 0.047 | 59.172 | 0.100 | 0.013 | 6.67E-14 | 0.054 | 0.206 | 0.79 |
| rs2231142 | 4:89052323 | T | G | 0.094 | 584.470 | 0.220 | 0.009 | 4.44E-116 | -0.099 | 0.209 | 0.64 |
| rs7654258 | 4:10404512 | T | C | 0.085 | 98.010 | -0.099 | 0.010 | 1.09E-19 | 0.002 | 0.198 | 0.99 |
| rs675209 | 6:7102084 | C | T | 0.269 | 103.252 | -0.063 | 0.006 | 1.38E-21 | 0.180 | 0.119 | 0.13 |
| rs729761 | 6:43804571 | G | T | 0.273 | 53.313 | 0.046 | 0.006 | 3.05E-12 | 0.022 | 0.122 | 0.85 |
| rs1178977 | 7:72857049 | G | A | 0.189 | 52.510 | -0.050 | 0.007 | 6.68E-12 | -0.143 | 0.145 | 0.32 |
| rs2941484 | 8:76478768 | T | C | 0.432 | 79.372 | 0.049 | 0.006 | 3.91E-17 | 0.013 | 0.111 | 0.91 |
| rs10761587 | 10:52649193 | C | T | 0.116 | 46.420 | -0.062 | 0.009 | 9.58E-11 | 0.117 | 0.160 | 0.47 |
| rs1171614 | 10:61469538 | C | T | 0.242 | 108.629 | 0.074 | 0.007 | 6.48E-23 | 0.166 | 0.150 | 0.27 |
| rs2078267 | 11:64334114 | T | C | 0.469 | 174.777 | -0.078 | 0.006 | 8.73E-36 | -0.047 | 0.111 | 0.68 |
| rs642803 | 11:65560620 | T | C | 0.457 | 63.409 | -0.043 | 0.005 | 4.51E-14 | -0.008 | 0.113 | 0.94 |
| rs3741414 | 12:57844049 | T | C | 0.195 | 102.878 | -0.071 | 0.007 | 9.79E-22 | -0.011 | 0.130 | 0.93 |
| rs653178 | 12:112007756 | T | C | 0.466 | 44.444 | -0.036 | 0.005 | 2.45E-10 | -0.067 | 0.112 | 0.55 |
| rs1394125 | 15:76158983 | A | G | 0.342 | 46.586 | 0.043 | 0.006 | 9.78E-11 | -0.021 | 0.127 | 0.87 |
| rs6598541 | 15:99271135 | G | A | 0.345 | 59.588 | -0.044 | 0.006 | 5.20E-13 | -0.187 | 0.116 | 0.11 |
| rs7193778 | 16:69563890 | T | C | 0.138 | 35.395 | -0.047 | 0.008 | 2.36E-08 | -0.043 | 0.161 | 0.79 |
| rs7224610 | 17:53364788 | A | C | 0.431 | 47.736 | -0.038 | 0.006 | 4.74E-11 | -0.088 | 0.112 | 0.43 |

Abbreviations: SNP: single nucleotide polymorphism; Chr: chromosome; EA: effect Allele; OA: other Allele; EFA: effect allele frequency; SE: standard error.

**Table S2.** List of SNPs used as instrumental variables for malignant neoplasm of cervix uteri.

| SNP | Chr: Position | EA | OA | EAF | *F*-Statistic | Association with exposure | | | Association with outcome | | |
| --- | --- | --- | --- | --- | --- | --- | --- | --- | --- | --- | --- |
|  |  |  |  |  |  | Beta | SE | *P*-value | Beta | SE | *P*-value |
| rs10927679 | 1:15118336 | T | C | 0.597 | 18.454 | -0.308 | 0.072 | 1.74E-05 | -0.004 | 0.006 | 0.56 |
| rs1153738 | 2:181998662 | A | G | 0.226 | 17.483 | 0.337 | 0.081 | 2.90E-05 | 0.001 | 0.006 | 0.84 |
| rs690037 | 3:16354161 | T | C | 0.514 | 16.513 | -0.289 | 0.071 | 4.83E-05 | -0.001 | 0.005 | 0.83 |
| rs1021549 | 13:43709745 | G | A | 0.039 | 16.927 | 0.625 | 0.152 | 3.88E-05 | 0.018 | 0.041 | 0.67 |
| rs1254837 | 14:61955518 | A | G | 0.950 | 17.733 | -0.584 | 0.139 | 2.54E-05 | -0.001 | 0.010 | 0.93 |

**Table S3.** List of SNPs used as instrumental variables for squamous cell neoplasms and carcinoma of cervix.

| SNP | Chr: Position | EA | OA | EAF | *F*-Statistic | Association with exposure | | | Association with outcome | | |
| --- | --- | --- | --- | --- | --- | --- | --- | --- | --- | --- | --- |
|  |  |  |  |  |  | Beta | SE | *P*-value | Beta | SE | *P*-value |
| rs6762937 | 3:86005981 | A | G | 0.561 | 17.107 | -0.424 | 0.102 | 3.53E-05 | -0.005 | 0.006 | 0.38 |
| rs11235209 | 11:87571751 | T | C | 0.615 | 18.409 | 0.473 | 0.110 | 1.78E-05 | 0.004 | 0.006 | 0.55 |
| rs277828 | 13:109041537 | A | C | 0.169 | 20.293 | -0.713 | 0.158 | 6.64E-06 | 0.011 | 0.007 | 0.13 |
| rs17180317 | 18:71076521 | G | A | 0.277 | 16.596 | 0.441 | 0.108 | 4.62E-05 | 0.011 | 0.007 | 0.13 |

**Table S4.** List of SNPs used as instrumental variables for adenocarcinomas of cervix.

| SNP | Chr: Position | EA | OA | EAF | *F*-Statistic | Association with exposure | | | Association with outcome | | |
| --- | --- | --- | --- | --- | --- | --- | --- | --- | --- | --- | --- |
|  |  |  |  |  |  | Beta | SE | *P*-value | Beta | SE | *P*-value |
| rs4570464 | 1:211030845 | C | A | 0.105 | 16.516 | 0.670 | 0.165 | 4.82E-05 | 0.006 | 0.008 | 0.47 |
| rs10123429 | 9:7818641 | A | C | 0.462 | 17.047 | 0.500 | 0.121 | 3.65E-05 | 0.001 | 0.006 | 0.91 |
| rs13300651 | 9:108611181 | C | A | 0.048 | 17.624 | 0.903 | 0.215 | 2.69E-05 | 0.071 | 0.039 | 0.08 |
| rs16908748 | 11:11043410 | G | A | 0.184 | 16.514 | -0.728 | 0.179 | 4.83E-05 | -0.003 | 0.007 | 0.66 |
| rs4245124 | 11:133728779 | C | A | 0.642 | 18.862 | -0.521 | 0.120 | 1.41E-05 | -0.008 | 0.007 | 0.28 |
| rs17263433 | 16:54591926 | C | T | 0.037 | 19.246 | 1.043 | 0.238 | 1.15E-05 | 0.042 | 0.022 | 0.07 |

**Table S5.** List of SNPs used as instrumental variables for carcinoma in situ of cervix uteri.

| SNP | Chr: Position | EA | OA | EAF | *F*-Statistic | Association with exposure | | | Association with outcome | | |
| --- | --- | --- | --- | --- | --- | --- | --- | --- | --- | --- | --- |
|  |  |  |  |  |  | Beta | SE | *P*-value | Beta | SE | *P*-value |
| rs12125759 | 1:37654332 | T | C | 0.263 | 17.977 | -0.148 | 0.035 | 2.24E-05 | 0.001 | 0.007 | 0.92 |
| rs6665740 | 1:81647312 | C | A | 0.713 | 19.863 | 0.152 | 0.034 | 8.32E-06 | 0.005 | 0.007 | 0.43 |
| rs11677276 | 2:217832226 | A | G | 0.482 | 17.044 | 0.125 | 0.030 | 3.65E-05 | 0.006 | 0.006 | 0.40 |
| rs1005147 | 3:26180987 | A | G | 0.032 | 17.368 | 0.327 | 0.078 | 3.08E-05 | 0.000 | 0.025 | 1.00 |
| rs6784002 | 3:108541848 | G | A | 0.507 | 19.525 | -0.133 | 0.030 | 9.93E-06 | 0.003 | 0.005 | 0.64 |
| rs6801770 | 3:175451941 | A | G | 0.023 | 16.506 | 0.368 | 0.091 | 4.85E-05 | 0.005 | 0.014 | 0.75 |
| rs13117312 | 4:47132770 | C | A | 0.055 | 19.320 | -0.318 | 0.072 | 1.11E-05 | 0.004 | 0.012 | 0.78 |
| rs3822512 | 5:172165054 | A | C | 0.449 | 20.609 | -0.138 | 0.030 | 5.63E-06 | 0.010 | 0.006 | 0.10 |
| rs465498 | 5:1325688 | G | A | 0.482 | 26.606 | -0.155 | 0.030 | 2.49E-07 | 0.002 | 0.005 | 0.76 |
| rs751138 | 5:41241508 | A | G | 0.155 | 18.100 | -0.185 | 0.043 | 2.10E-05 | -0.005 | 0.009 | 0.60 |
| rs17085831 | 6:154985994 | G | A | 0.060 | 18.987 | 0.258 | 0.059 | 1.32E-05 | 0.016 | 0.026 | 0.56 |
| rs9293930 | 6:73456375 | G | A | 0.850 | 21.294 | -0.186 | 0.040 | 3.94E-06 | 0.004 | 0.008 | 0.63 |
| rs13242051 | 7:52006201 | T | G | 0.249 | 24.417 | 0.167 | 0.034 | 7.76E-07 | -0.003 | 0.006 | 0.63 |
| rs13250152 | 8:33001909 | T | C | 0.045 | 27.654 | -0.429 | 0.082 | 1.45E-07 | 0.031 | 0.022 | 0.18 |
| rs10119821 | 9:28185005 | G | A | 0.026 | 16.567 | 0.340 | 0.083 | 4.70E-05 | 0.045 | 0.023 | 0.07 |
| rs1197922 | 9:28004208 | G | A | 0.687 | 18.296 | 0.142 | 0.033 | 1.89E-05 | -0.001 | 0.006 | 0.83 |
| rs11026150 | 11:21658363 | T | C | 0.045 | 18.258 | -0.341 | 0.080 | 1.93E-05 | 0.027 | 0.025 | 0.31 |
| rs1526851 | 12:105492578 | A | G | 0.198 | 19.223 | -0.171 | 0.039 | 1.16E-05 | -0.011 | 0.007 | 0.11 |
| rs7971628 | 12:43959780 | T | G | 0.901 | 17.106 | -0.198 | 0.048 | 3.54E-05 | 0.002 | 0.007 | 0.79 |
| rs2793821 | 13:95179507 | G | T | 0.329 | 17.689 | 0.133 | 0.032 | 2.60E-05 | -0.008 | 0.006 | 0.19 |
| rs7492479 | 14:95740509 | C | T | 0.144 | 17.882 | 0.174 | 0.041 | 2.35E-05 | -0.016 | 0.008 | 0.08 |
| rs4811486 | 20:53945163 | T | C | 0.210 | 16.804 | -0.156 | 0.038 | 4.14E-05 | -0.002 | 0.007 | 0.82 |
| rs7059 | 20:410526 | T | C | 0.562 | 17.869 | -0.128 | 0.030 | 2.37E-05 | -0.008 | 0.006 | 0.23 |
| rs2253313 | 21:34815603 | G | T | 0.408 | 17.004 | -0.127 | 0.031 | 3.73E-05 | 0.001 | 0.006 | 0.90 |

**Table S6.** List of SNPs used as instrumental variables for other benign neoplasm of uterus: cervix uteri.

| SNP | Chr: Position | EA | OA | EAF | *F*-Statistic | Association with exposure | | | Association with outcome | | |
| --- | --- | --- | --- | --- | --- | --- | --- | --- | --- | --- | --- |
|  |  |  |  |  |  | Beta | SE | *P*-value | Beta | SE | *P*-value |
| rs12729804 | 1:211724416 | T | C | 0.052 | 19.060 | 0.837 | 0.192 | 1.27E-05 | 0.000 | 0.010 | 0.97 |
| rs6926485 | 6:112202408 | C | T | 0.407 | 17.392 | 0.446 | 0.107 | 3.04E-05 | -0.006 | 0.006 | 0.34 |
| rs4741546 | 9:15846114 | T | C | 0.466 | 16.509 | -0.446 | 0.110 | 4.84E-05 | -0.004 | 0.006 | 0.51 |


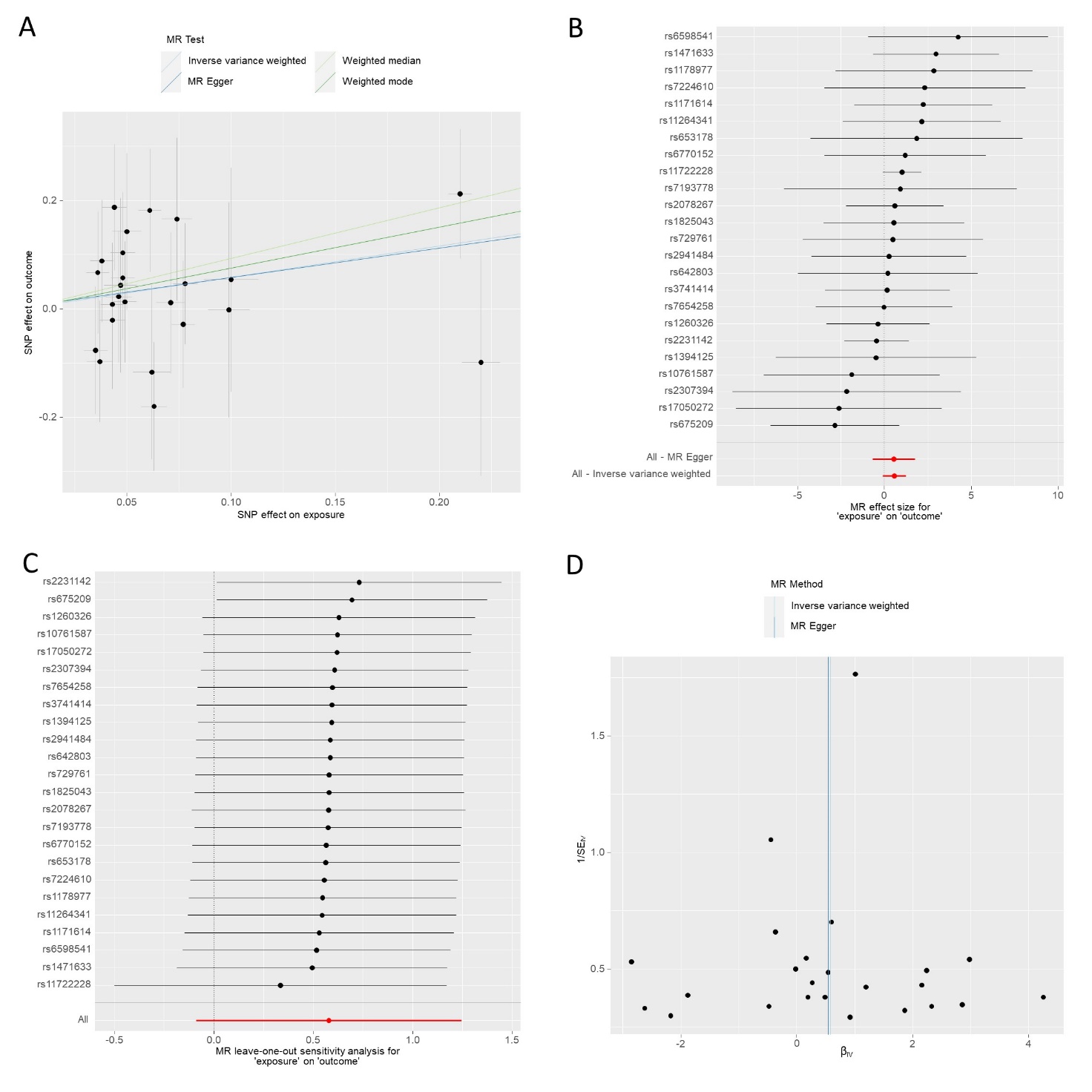
 **Figure S1.** Scatter plot (A), forest plot (B), sensitivity analysis (C) and funnel plot (D) of the causal effect of urate on risk of squamous cell neoplasms and carcinoma of cervix.


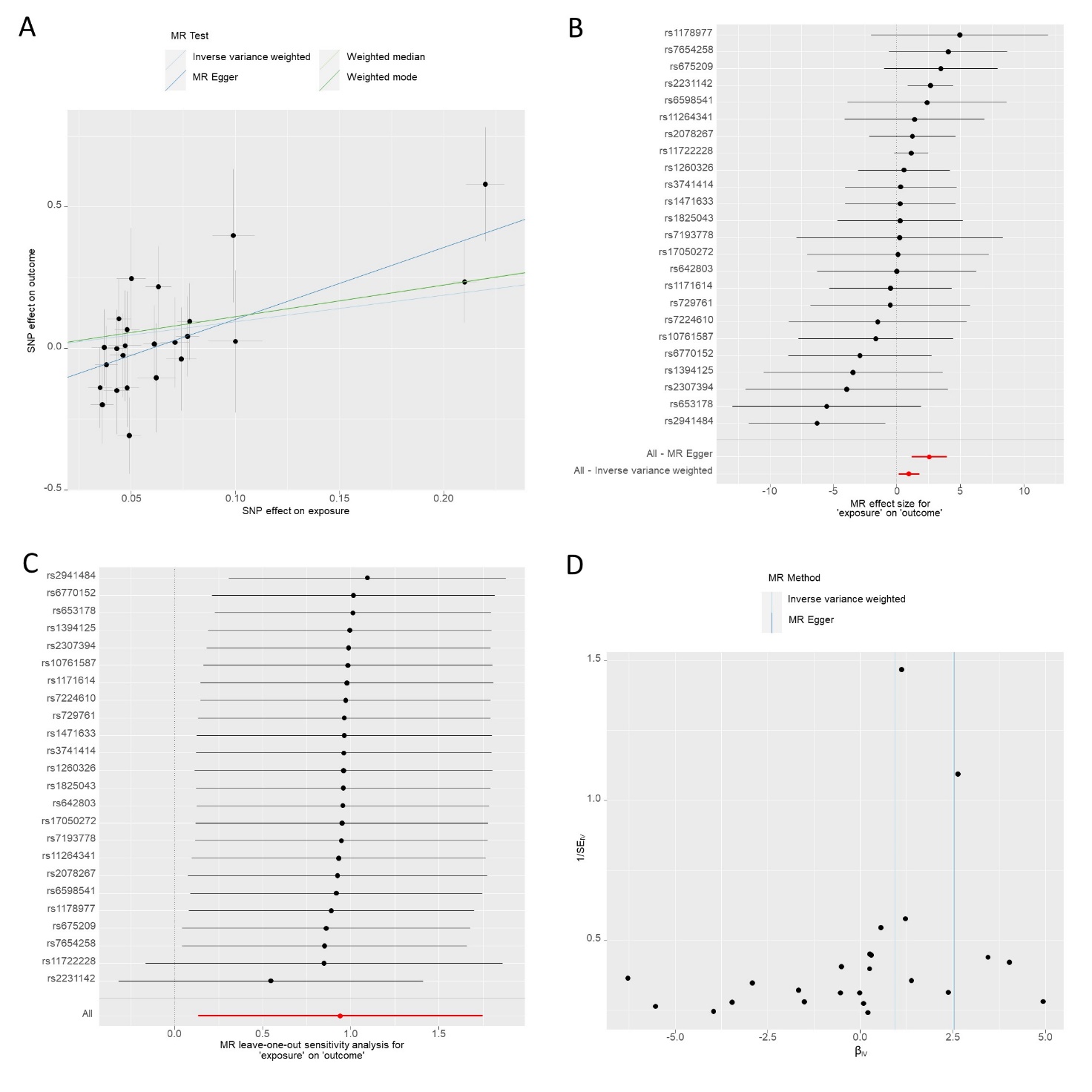
 **Figure S2.** Scatter plot (A), forest plot (B), leave-one-out analysis (C) and funnel plot (D) of the causal effect of urate on risk of adenocarcinomas of cervix.


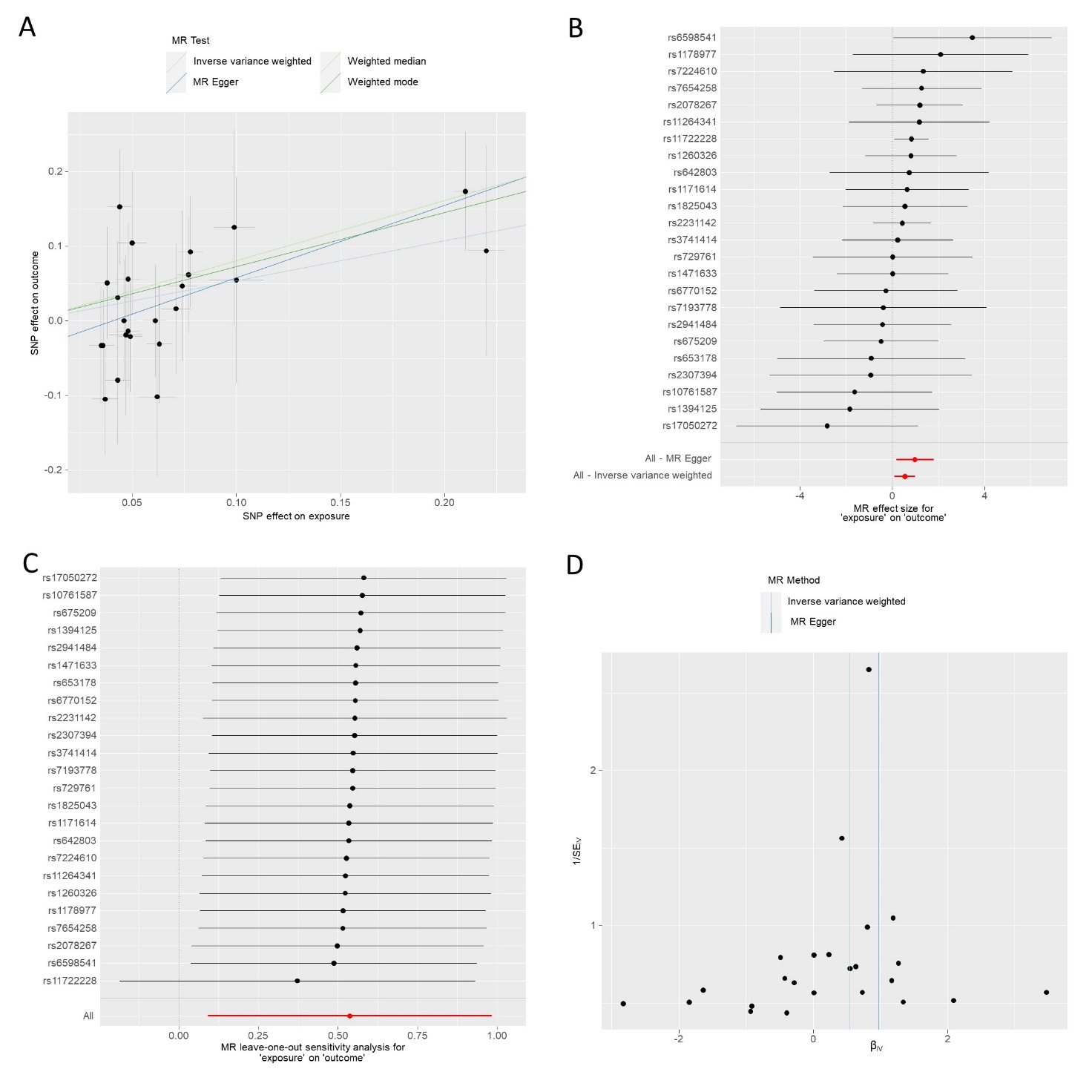


**Figure S3.** Scatter plot (A), forest plot (B), sensitivity analysis (C) and funnel plot (D) of the causal effect of urate on risk of malignant neoplasm of uterus: cervix uteri.


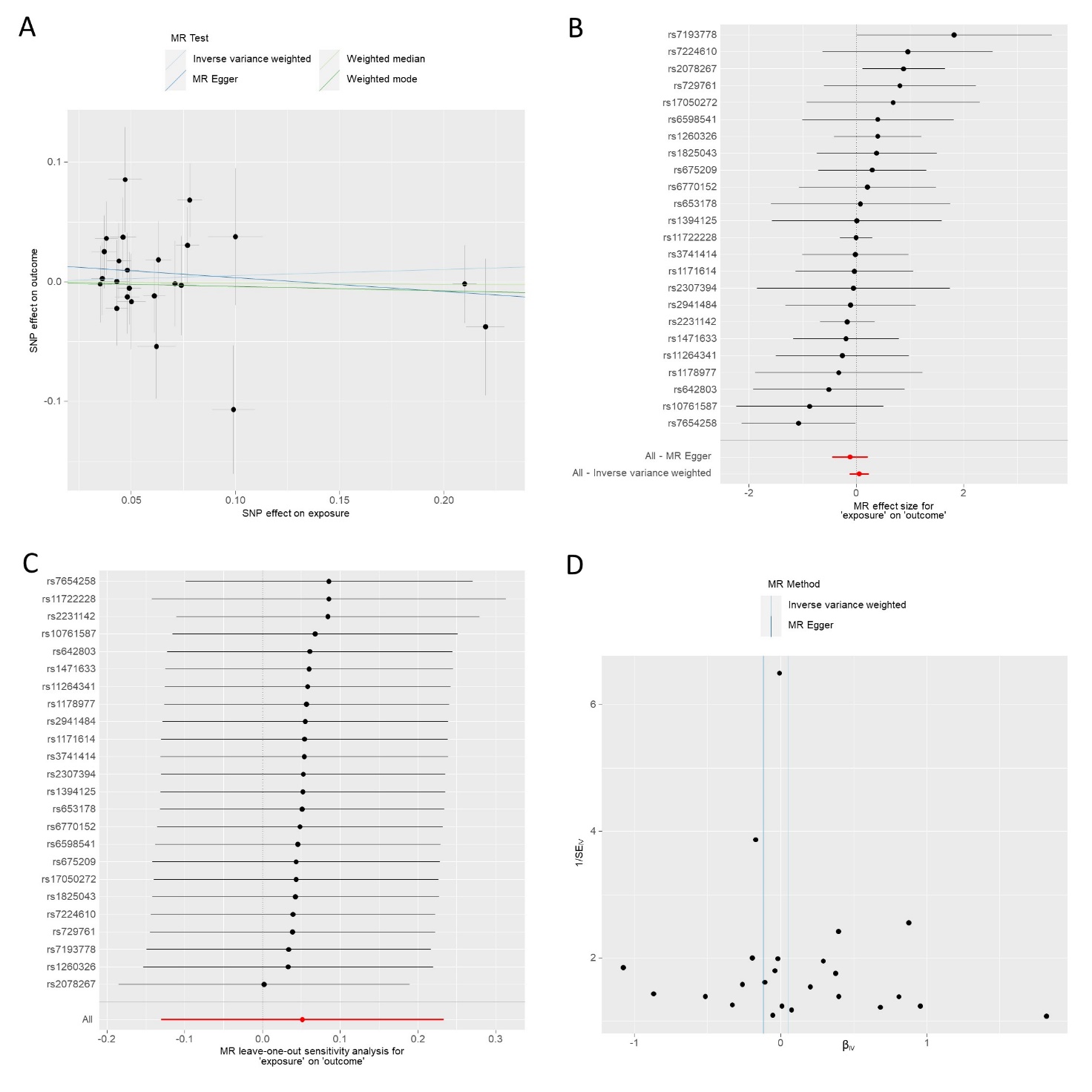


**Figure S4.** Scatter plot (A), forest plot (B), sensitivity analysis (C) and funnel plot (D) of the causal effect of urate on risk of carcinoma in situ of cervix uteri.


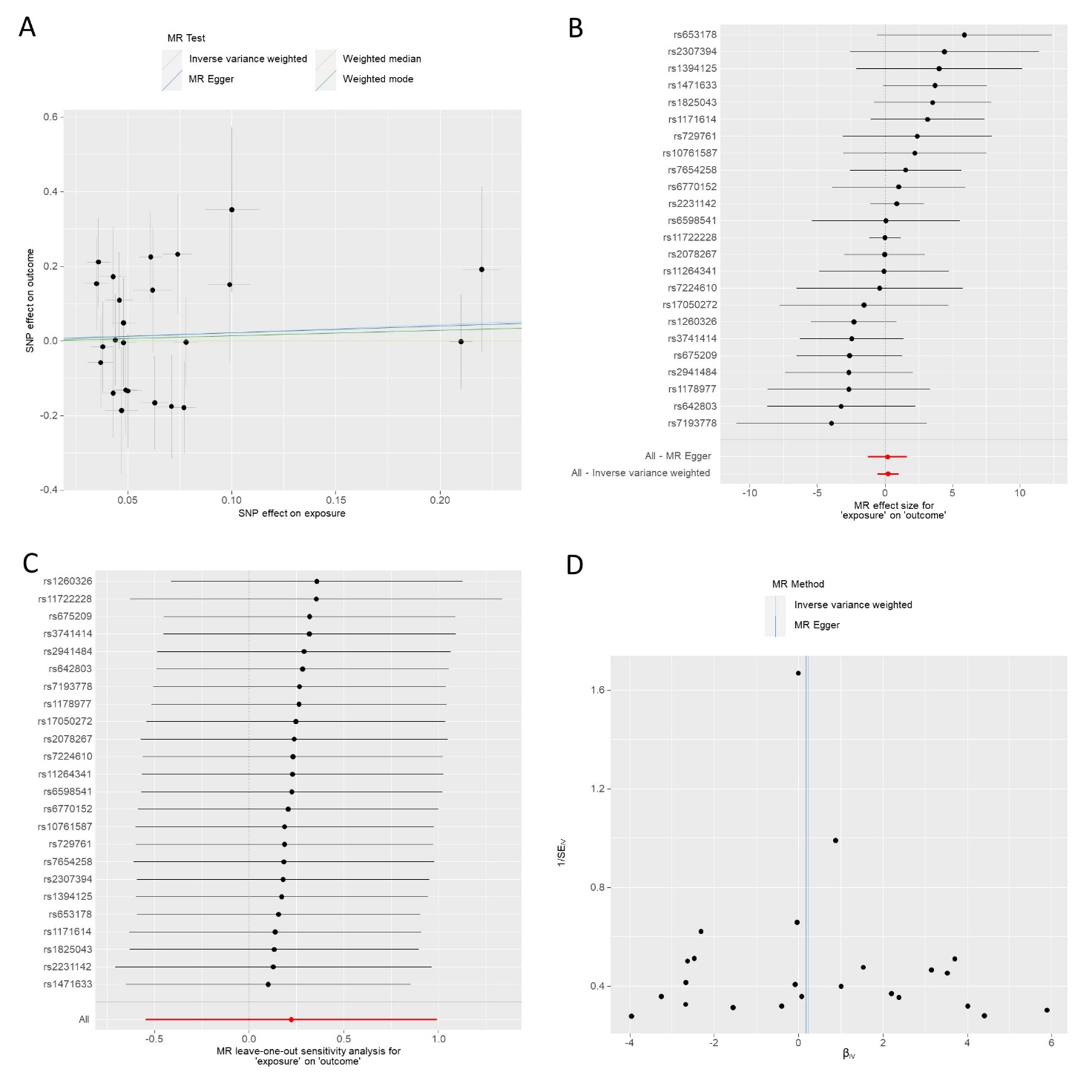


**Figure S5.** Scatter plot (A), forest plot (B), sensitivity analysis (C) and funnel plot (D) of the causal effect of urate on risk of other benign neoplasm of uterus: cervix uteri.
